# Comparative Study of Oxidative Structural Modifications of Unadsorbed and Adsorbed Proteins in Whey Protein Isolate-Stabilized Oil-in-Water Emulsions under the Stress of Primary and Secondary Lipid Oxidation Products

**DOI:** 10.3390/foods10030593

**Published:** 2021-03-11

**Authors:** Jiaxin Chen, Jinhai Zhao, Baohua Kong, Qian Chen, Qian Liu, Chengguo Liu

**Affiliations:** 1 College of Food Science, Northeast Agricultural University, Harbin 150030, China; chenjiaxin993523@163.com (J.C.); kongbh63@hotmail.com (B.K.); chenqianego7@126.com (Q.C.); 2 Institute for Advanced Technology, Heilongjiang Academy of Sciences, Harbin 150001, China; zjh345729635@163.com; 3 National Dairy Engineering & Technology Research Center, Heilongjiang Green Food Science & Research Institute, Harbin 150028, China; 4 Sharable Platform of Large-Scale Instruments & Equipments, Northeast Agricultural University, Harbin 150030, China

**Keywords:** whey protein isolate, oil-in-water emulsion, protein oxidative modification, lipid oxidation products, protein structure

## Abstract

The impact of typical primary or secondary lipid oxidation (LPO) products, selected as linoleic acid 13-hydroperoxide (13-HPODE) and malondialdehyde (MDA), on the structural modification of unadsorbed or adsorbed proteins in whey protein isolate (WPI)-stabilized oil-in-water (O/W) emulsions during storage up to 48 h at 37 °C in the dark was investigated. The results showed that either 13-HPODE and MDA could lead to structural modifications of unadsorbed or adsorbed proteins with a concentration-dependent manner and time relationship, respectively. Moreover, higher levels of MDA rendered a higher degree of oxidative modifications of WPI than 13-HPODE, indicated by the higher protein carbonyl contents and N’-formyl-L-kynurenine (NFK) and lower fluorescence intensity. Additionally, adsorbed proteins were more easily oxidized by LPO products than unadsorbed proteins. Overall, our results indicated that the formation of secondary LPO products and the protein position were crucial factors to increase the degree of oxidative modifications of WPI in O/W emulsion systems.

## 1. Introduction

Natural proteins, not only extracted from animal sources (such as whey, gelatin, albumin, sodium caseinate, and bovine serum albumin, etc.), but also extracted from plant sources (such as soybean, pea, chickpea, zein, and faba bean, etc.) are commonly acted as food emulsifiers to prepare and stabilize oil-in-water (O/W) emulsions [1,2]. Moreover, enrichment of numerous types of emulsion foods with polyunsaturated fatty acids (PUFAs) has been favoured more and more by consumers all over the world, mainly due to their potential health benefits, especially for preventing the occurrence of cardiovascular diseases [3]. However, PUFAs are easily prone to lipid oxidation (LPO) mainly due to their multiple unsaturated double bonds, which subsequently leads to the quality deterioration of emulsion foods [4]. LPO is a highly complex free radical chain reaction, which could promote the generation of several chemical reaction products. The odourless and tasteless hydroperoxides are recognized as primary reaction products of the LPO, and they further decompose to secondary reaction products such as saturated aldehydes, unsaturated aldehydes, and dialdehydes with increasing storage time [5]. As far as we know, these LPO products, especially for hydroperoxides and malondialdehyde (MDA), could easily and directly react with proteins and then accelerate the formation of various reaction adducts, which not only lead to the deterioration of textural properties and sensorial evaluation of emulsion foods, but also result in the loss of nutritive values [6,7,8]. For instance, Wu et al. [9] indicated that 13-hydroperoxyoctadecadienoic acid (13-HPODE) could significantly enhance the carbonyl content and decrease the sulfhydryl content of rice protein in a dose-dependent manner, as well as promote protein aggregation via disulphide bonds. Wu et al. [10] also reported that MDA could efficiently react with ε-amino and sulfhydryl groups of soybean protein, which are closely associated with the structural changes via non-disulfide covalent bonds. Moreover, Zhu et al. [11] suggested that MDA could lead to the obvious droplet flocculation of chicken sarcoplasmic protein-based O/W emulsion, which was mainly attributed to protein aggregation via disulfide bonds. Mestdagh et al. [12] also indicated that light-induced oxidation could significantly promote the formation of conjugated diene hydroperoxides of whey protein-based O/W emulsion, which subsequently resulted in protein aggregation when measured by using tryptophan fluorescence.

However, O/W emulsion belongs to a typical system that consists of oil droplets dispersed in an aqueous phase [13]. In the fabrication of O/W emulsions, proteins are either adsorbed at the surface of oil droplets (termed adsorbed protein) or dispersed in the aqueous phase (termed unadsorbed protein). Thus, the position at which proteins are located in interfacial layers or in aqueous phase has to be seriously taken in account when LPO occur in multiphase systems under various environments/conditions. Zhu et al. [4] found that compared with unadsorbed proteins, adsorbed proteins were much more easily oxidized induced by LPO. Gumus et al. [14] also indicated that unadsorbed proteins could positively act as better antioxidants and retard LPO and then inhibit the related LPO products to reach the surfaces of lipid droplets. Furthermore, Berton et al. [15] reported that adsorbed proteins underwent a higher degree of structural modifications during the whole oxidation of β-lactoglobulin-stabilized O/W emulsions; nevertheless, unadsorbed proteins in the aqueous phase were much less prone to oxidized damage, which was clearly verified by unchanged protein solubility and no protein aggregation. Conversely, the results provided by intrinsic tryptophan fluorescence scanning in situ have suggested that decreased fluorescent intensity was a distinct consequence to indicate the structural modifications of the proteins induced by LPO during the storage of O/W emulsions [16,17,18,19]. Therefore, the protein position in O/W emulsions plays a crucial role in the evaluation of the protein modifications which are induced by LPO. However, there is little information as regards to the influence of different concentrations of LPO products, especially for primary and secondary products, on the structural changes of unadsorbed and adsorbed proteins from O/W emulsions. Our previous work successfully established an efficient and eco-friendly extraction method for adsorbed proteins from emulsions [20], which could provide greater convenience for our further research.

The objective of our present study was to investigate the effect of different concentrations (0, 1, 10, 100, 1000 μmol/L emulsion) of 13-HPODE or MDA (selected as typical primary and secondary LPO products) on the structural characteristics of unadsorbed and adsorbed proteins from a whey protein isolate (WPI)-stabilized O/W emulsion during 24 and 48 h incubation at 37 °C in the dark. Fully hydrogenated coconut oil (melting point, 33.3 °C) was used as the oil phase to produce the O/W emulsions to ensure that no extra LPO products would be produced during the incubation period. In addition, the structural characteristics, such as the carbonyl content, N’-formyl-L-kynurenine (NFK), intrinsic fluorescence, and electrophoresis of extracted unadsorbed and adsorbed proteins, were monitored by various analytical methods.

## 2. Materials and Methods

### 2.1. Materials

Native WPI (Item No. 9410) was obtained from Hilmar Ingredients Co. Ltd. (Hilmar, CA, USA); the approximate composition was as follows: 89.0% protein, 4.7% moisture, 1.3% fat, 2.7% ash, and 0.1% lactose (based on the total weight basis). Tween 20 and 1,1,3,3-tetraethoxypropane (TMP) were purchased from Aladdin Reagent Co. Ltd. (Shanghai, China). Linoleic acid and lipoxidase (soybean) were purchased from Yuanye Bio-Technology Co. Ltd. (Shanghai, China). Fully hydrogenated coconut oil (melting point, 33.3 °C) was purchased from Global Natural Perfume Co. Ltd. (Ji’an City, Jiangxi, China). All the other reagents were of analytical grade.

### 2.2. Preparation of Primary LPO Products (13-HPODE)

The linoleic acid 13-HPODE was prepared according to the method of Iacazio et al. [21]. Briefly, 280 mg linoleic acid was transferred into a 60 mL Schlenck tube placed in an ice bath (0–4 °C). Then, 10.0 mL of sodium borate buffer (0.1 M, pH 9.0) was mixed with the linoleic acid and stirred for 5 min at a speed of 500 rpm via an electric stirrer (MYP2011-100, IKA-Werke GmbH & Co. Ltd., Guangzhou, China). After that, 10.0 mg of the soybean lipoxidase powder (10 U/mg) was added to initiate the reaction, and pure oxygen was used to pressurize the reaction vessel. Then, the stirring speed was accelerated to 1000 rpm, and the whole reaction progress lasted for 1 h. Finally, 50.0 μL of the above solution was diluted in 100 mL ethanol to measure the concentration of hydroperoxide by using a ultraviolet-visible (UV-vis) spectrophotometer (T6, Beijing Purkinje General Instrument Co., Ltd. Beijing, China) at 234 nm with a molar extinction coefficient value of 25,000 M^−1^ cm^−1^.

### 2.3. Preparation of Secondary LPO Products (MDA)

The MDA solution was prepared according to the method of Adams et al. [22] with a slight modification. Briefly, 1100 mg TMP was transferred into a 100 mL glass beaker and mixed with 50.0 mL 0.1 M HCl. Then, the solution was kept at 50 °C and stirred for 60 min at a speed of 500 rpm by using a heatable magnetic stirrer (C-MAG-HS10, IKA-Werke GmbH & Co. Ltd., Guangzhou, China). After that, pH of the solution was adjusted to 7.0 by 6 M NaOH. Finally, the MDA solution was diluted to 10^−5^ by sodium phosphate buffer (10 mM, pH 7.0) to estimate the concentration of MDA using a UV-vis spectrophotometer (T6, Beijing Purkinje General Instrument Co., Ltd. Beijing, China) at 267 nm with a molar extinction coefficient value of 31,500 M^−1^ cm^−1^.

### 2.4. Emulsion Preparation

Native WPI powder was gently dispersed in phosphate buffer (10 mM, pH 7.0) and stirred magnetically at 500 rpm for 2 h and then stored overnight at 4 °C to produce a fully hydrated stock WPI solution (2.0%, *w*/*v*). In order to avoid droplet flocculation of WPI-stabilized O/W emulsions under acidic conditions since the isoelectric point of WPI is approximately 4.6 [23], the neutral pH of the WPI solution was selected in our experiment. Moreover, as indicated by the results of Chen et al. [20], the O/W emulsion prepared with 2.0% (*w*/*v*) WPI solution showed the highest physical stability during the storage.

Before emulsion preparation, the fully hydrogenated coconut oil and the WPI stock solution were kept at 40 °C by a water-bath heating method to prevent coconut oil solidification. To prepare the O/W emulsions, 5.0 mL each of coconut oil was expeditiously mixed with different concentrations of 13-HPODE solution or MDA solution (0, 1, 10, 100, and 1000 mmol/L emulsion) by using a vortex oscillator (Vortex-Genie2, Scientific Industries, Bohemia, NY, USA) at a speed of “5” for 10 s. Then, 45.0 mL of the WPI solution was mixed with the above coconut oils by using a high-shear homogenizer (IKA T18 basic, IKA-Werke GmbH & Co., Staufen, Germany) at 13,500 rpm for 2 min. After that, the coarse emulsions were further homogenized by an ultrasonic probe (FS-100T, Shanghai, China) according to the method of Chen et al. [20]. Sodium azide (0.02%, *w*/*v*) was added to the emulsions to prevent microbial growth. The emulsions were quickly transferred to 50 mL sealed plastic tubes and stored in the dark at 37 °C for pending analysis. The indicators of protein oxidation in each of the O/W emulsions were measured at 24 and 48 h.

### 2.5. Recovery of Unadsorbed and Adsorbed Proteins

The extraction methods of unadsorbed and adsorbed proteins followed the same procedures as those used by Chen et al. [20].

### 2.6. Determination of Protein Concentrations

The concentrations of unadsorbed and adsorbed proteins were determined via BCA protein assay kits (Item No. PC0020, Solarbio Life Scientifics, INC., Beijing, China) and expressed as mg/mL emulsion. The detailed procedure was according to the operation instruction. Bovine serum albumin (BSA) was applied as the standard.

### 2.7. Measurement of the Protein Carbonyl Content

The protein carbonyl contents of unadsorbed and adsorbed proteins were determined according to the method of Levine et al. [24] with some modifications. Briefly, 0.5 mL each of unadsorbed and adsorbed protein solution (2.0 mg/mL) was incubated with 0.5 mL 2, 4-Dinitrophenylhydrazin (DNPH) (10 mM) solution in a 2.0 mL centrifuge tube for 60 min at 25 °C and vortexed every 15 min for 5 s at speed of “5” via a vortex mixer (3030A, Scientific Industries, INC., Bohemia). After that, 0.5 mL trichloroacetic acid (20%, *v*/*v*) was added in the samples, and then the samples were centrifuged at 11,000× *g* for 5 min to precipitate proteins. The precipitate pellets were washed three times with 0.5 mL ethanol-ethyl acetate (1:1, *v*/*v*) to remove free reagent. Then, the precipitate pellets were redissolved in 1.0 mL guanidine solution (6 M) within 15 min at 37 °C. Finally, the samples were transferred to a 200 μL quartz cuvette (path, 1 cm) to measure the contents of carbonyl by using a UV-vis spectrophotometer (T6, Beijing Purkinje General Instrument Co., Ltd. Beijing, China) at 370 nm with a molar absorption coefficient of 22,000 M^−1^ cm^−1^. The contents of protein carbonyl were expressed as μmol/g protein.

### 2.8. NFK

The levels of NFK of unadsorbed and adsorbed proteins were measured via a fluorescence spectrophotometer (F-4500 model, Hitachi, Tokyo, Japan) according to the procedure of Mestdagh et al. [12] with some modifications. Briefly, each of the unadsorbed and adsorbed protein solutions were diluted to 0.1 mg/mL by 10 mM phosphate buffer (pH 7.0), and then transferred to a quartz cuvette. The excitation wavelength was set at 330 nm, and the fluorescence intensity at 440 nm was recorded. The slit width was 5 nm, and the voltage was 700 mV.

### 2.9. Intrinsic Tryptophan Fluorescence

The intrinsic tryptophan fluorescence of the unadsorbed and adsorbed proteins was determined by using a fluorescence spectrophotometer (F-4500 model, Hicathi, Tokyo, Japan) according to the method of Chen et al. [20].

### 2.10. Sodium Dodecyl Sulfate Polyacrylamide Gel Electrophoresis (SDS-PAGE)

SDS-PAGE procedures of unadsorbed and adsorbed proteins were performed according to the method of Liu et al. [25] under non-reducing conditions.

### 2.11. Statistical Analysis

All experimental data were measured in triplicate, and three independent tests were conducted. The data are shown as the means ± standard deviations (SD) analyzed via the Statistix 8.1 software package (Analytical Software, St. Paul, MN, USA). The figures were drawn by Sigmaplot 12.5 (Graphics Software, San Jose, CA, USA). One-way analysis of variance (ANOVA) was implemented to analyse the significance of the main effects (*p* < 0.05) between means using Tukey’s multiple comparison test. Moreover, the bivariate correlations between the levels of 13-HPODE/MDA and the protein carbonyl/NFK of unadsorbed or adsorbed proteins were established by using SPSS statistics software version 25 (IBM software, Armonk, NY, USA), and the related coefficient of each bivariate correlation was expressed as “Φ”. Additionally, different uppercase letters in the same column indicate significant differences (*p* < 0.05) in each table.

## 3. Results and Discussion

### 3.1. Concentrations of Unadsorbed and Adsorbed Proteins

The concentrations of unadsorbed and adsorbed proteins extracted from the O/W emulsions after 24 or 48 h inoculation time are shown in Table 1 and Table 2. After 24 h inoculation time, there was no significant difference (*p* > 0.05) in the concentrations of unadsorbed proteins when the addition levels of 13-HPODE or MDA increased from 0 to 10 μmol/L. However, the concentrations of unadsorbed proteins extracted from the emulsions with relatively higher addition levels (100 and 1000 μmol/L) of 13-HPODE or MDA were significantly lower (*p* < 0.05), and the lowest concentrations of unadsorbed proteins were found in the emulsion with 1000 μmol/L addition levels of MDA (*p* < 0.05). For example, the concentrations of unadsorbed proteins decreased by 4.39% and 7.61% when the levels of 13-HPODE and MDA increased from 0 to 1000 μmol/L, respectively. Contrarily, the concentrations of the absorbed proteins showed an increasing trend with increasing addition levels of 13-HPODE or MDA, and the highest concentrations of the absorbed proteins were found in the emulsion with 1000 μmol/L MDA (*p* < 0.05). For instance, concentrations of adsorbed proteins increased by 15.99% and 20.89% when the levels of 13-HPODE and MDA increased from 0 to 1000 μmol/L, respectively. A similar trend was obtained in the inoculated emulsions after 48 h inoculation time.

The higher concentration of 13-HPODE or MDA could induce the oxidation stress of the natural proteins, simultaneously causing an unfolding of the protein spatial structure and exposure of hydrophobic amino acid residues [9,26]. The exposure of hydrophobic amino acid residues may enhance the hydrophobic interaction between the proteins and the lipid in the emulsions, causing a decrease in the unadsorbed proteins and an increase in the adsorbed proteins. MDA is an amphipathic molecule that can readily react with natural proteins, generating a wide variety of intra- and inter-molecular covalent adducts, causing a part of the MDA combined protein to transfer to the interface of the oil droplets [27,28]. In addition, the concentrations of unadsorbed proteins increased and those of adsorbed proteins decreased after 48 h inoculation compared with the emulsions inoculated for 24 h. This may be due to aggregation of the adsorbed proteins causing the proteins to be dropped from the oil-water interface [12,15].

### 3.2. Measurement of the Protein Carbonyl Content

As a practical indicator, protein carbonyl is widely used to indicate the extent of protein damage in the process of protein oxidation [29]. The carbonyl contents of unadsorbed and adsorbed proteins extracted from the O/W emulsions after 24 h inoculation are shown in Table 3. As indicated by the values of Φ, the carbonyl contents of unadsorbed and adsorbed proteins showed a positive correlation with the increasing addition levels of 13-HPODE or MDA. Relatively higher addition levels of 13-HPODE or MDA had a significant impact on the carbonyl contents of unadsorbed and adsorbed proteins (*p* < 0.05), and the highest carbonyl contents were found in the adsorbed proteins from the emulsions with 1000 μmol/L addition levels of MDA (*p* < 0.05). After 48 h inoculation, except for the higher carbonyl contents of the unadsorbed and adsorbed proteins, a similar increasing trend of the carbonyl contents was found in comparison with the emulsions inoculated for 24 h; the results are shown in Table 4.

In this study, the carbonyl contents of the unadsorbed and adsorbed proteins extracted from the emulsions with relatively higher addition levels of MDA were significantly higher than those in the emulsions with the same addition levels of 13-HPODE at both 24 and 48 inoculation times (*p* < 0.05). To some degree, this result proved that MDA could cause more serious protein oxidative damage than 13-HPODE at relatively higher addition levels. However, it is notable that MDA is one of the typical carbonyl compounds that contains two carbonyl groups [11]. When the proteins are attacked by MDA, the carbonyl groups may enter the nucleophilic side chain residues (such as cysteine, histidine, tryptophan, and lysine residues) of the proteins [30]. Moreover, the carbonyl groups also can be introduced into the proteins by the reaction between MDA with primary amino groups of the proteins [10]. Therefore, the higher carbonyl contents of the unadsorbed and adsorbed proteins extracted from the emulsions with relatively higher addition levels of MDA may also relate to the newly introduced carbonyl group contained in MDA itself.

### 3.3. Measurement of NFK

NFK is an intermediate generated from the oxidative stress of tryptophan and tryptophan residues via several reactions [31]. The NFK values of unadsorbed and adsorbed proteins extracted from the O/W emulsions after 24 and 48 h inoculation are shown in Table 5 and Table 6. As indicated by the values of Φ, the NFK of unadsorbed and adsorbed proteins showed a positive correlation with the increasing addition levels of 13-HPODE or MDA. After 24 h inoculation, the NFK of the unadsorbed and adsorbed proteins gradually increased with the increasing addition levels of the 13-HPODE or MDA. For the unadsorbed proteins, higher NFK of the unadsorbed proteins was observed in the emulsions with relatively higher addition levels of 13-HPODE or MDA (*p* < 0.05), and the highest NFK was found in the emulsion with 1000 μmol/L addition levels of MDA (*p* < 0.05). For the NFK of adsorbed proteins, there were no significant differences when the addition levels of the 13-HPODE less than 10 μmol/L (*p* > 0.05), and the highest NFK was shown in the emulsions with 1000 μmol/L MDA (*p* < 0.05). After 48 h inoculation time, the NFK of the unadsorbed and adsorbed proteins in all the inoculated emulsions increased. Furthermore, the NFK of the adsorbed proteins was obviously higher than that of the unadsorbed proteins especially in the emulsions with relatively higher addition levels of 13-HPODE or MDA (*p* < 0.05). The existence of NFK suggests that the oxidative degradation of tryptophan residues occurred in the proteins [32]. Dalsgaard et al. [33] indicated that the loss of milk protein tryptophan by photo-oxidation was well-correlated with the formation of NFK. Mestdagh et al. [12] also reported that the increasing trend of NFK in the illuminated emulsions was positively correlated with the extent of tryptophan degradation. Additionally, the increased NFK level was closely associated with the changes in the tertiary structure of proteins and the subsequent fragmentation of proteins [34].

### 3.4. Measurement of Intrinsic Tryptophan Fluorescence

**Figure 1:**
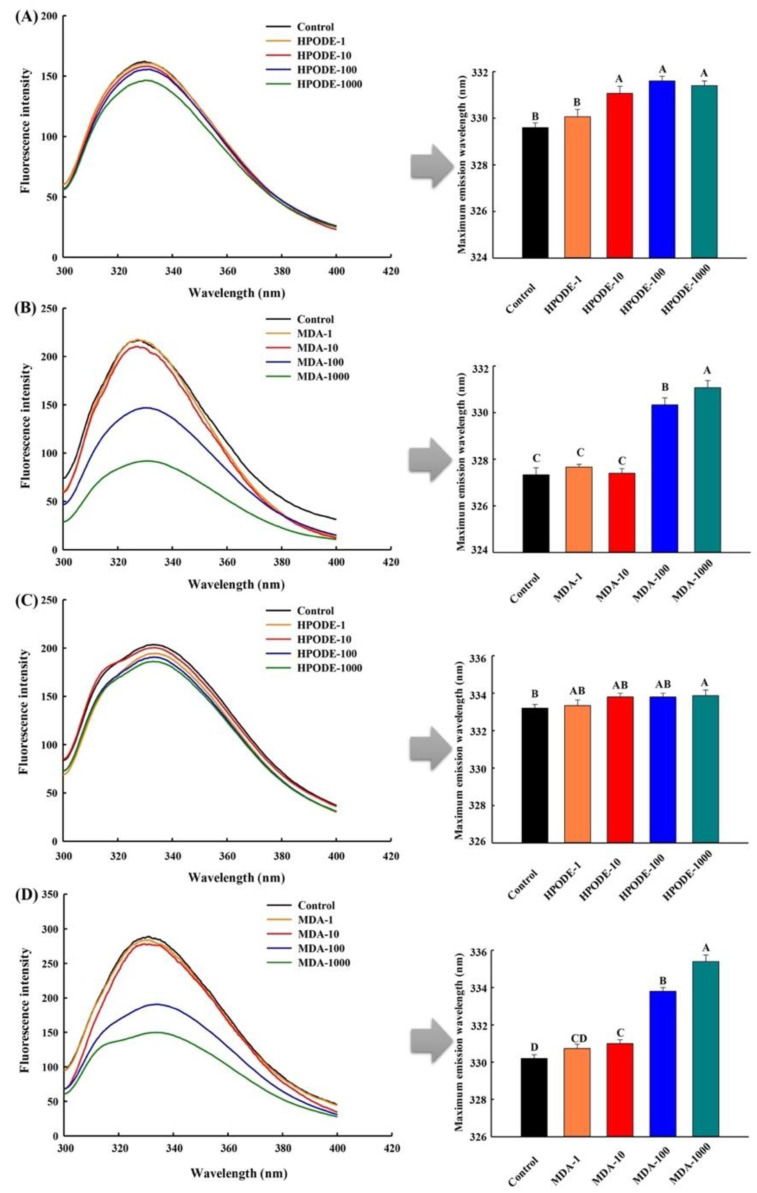
Intrinsic tryptophan fluorescence of the unadsorbed (**A**,**B**) and adsorbed proteins (**C**,**D**) extracted from O/W emulsions oxidized by different levels of HPODE and MDA (0, 1, 10, 100, and 1000 μmol/L emulsion) after 24 h incubation. Corresponding maximum emission wavelengths are shown in the right side.

The analysis of intrinsic tryptophan fluorescence has been normally used for the assessment of changes in the tertiary structure of proteins [35]. As shown in Figure 1, the fluorescence spectra of the unadsorbed and adsorbed proteins extracted from the O/W emulsions after 24 h inoculation showed a main peak in the wavelength range of 329–336 nm. Compared with the emulsions inoculated with 13-HPODE, the fluorescence intensity of unadsorbed and adsorbed proteins presented a dramatic decrease when the addition levels of MDA reached 100 and 1000 μmol/L (*p* < 0.05). The obvious disappearance of fluorescence was principally due to the following two reasons, one is the unfolding of the protein tertiary structure and the exposure of hydrophobic sites [36], the other is that MDA may bind to the other sites of the unfolding protein causing the destruction of tryptophan residues, changing microenvironment in which tryptophan residues are located [37]. Moreover, a red-shift phenomenon was observed in the maximum emission wavelengths of the unadsorbed and adsorbed proteins, especially in the emulsions with relatively higher addition levels of MDA (*p* < 0.05). This result might be due to the changes in the microenvironment of the protein via oxidation stress, which meant more tryptophan residues in the proteins were exposed in the polar environment [38].

**Figure 2:**
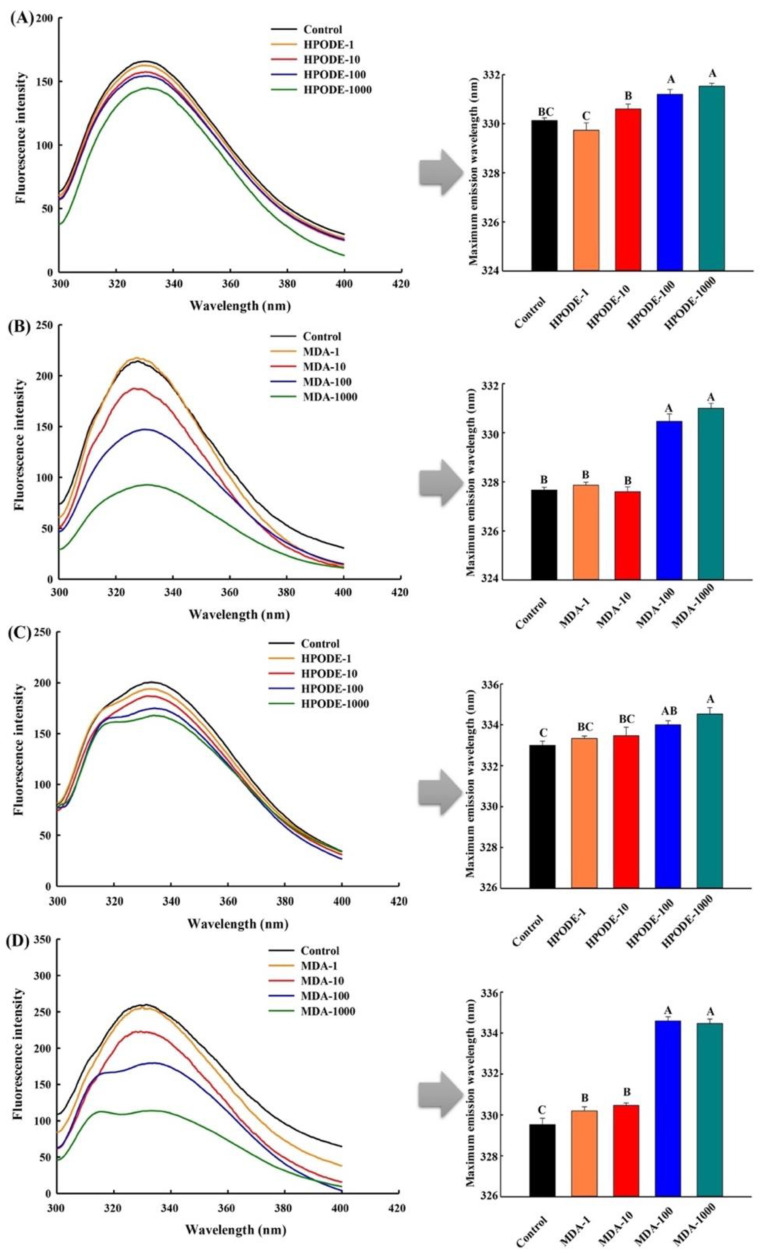
Intrinsic tryptophan fluorescence of the unadsorbed (**A**,**B**) and adsorbed proteins (**C**,**D**) extracted from O/W emulsions oxidized by different levels of HPODE and MDA (0, 1, 10, 100, and 1000 μmol/L emulsion) after 48 h incubation. Corresponding maximum emission wavelengths are shown in the right side.

As shown in Figure 2, the fluorescence intensity of the unadsorbed and adsorbed proteins extracted from the O/W emulsions showed a more evident decrease after 48 h inoculation. Moreover, similar changes were observed in the maximum emission wavelengths compared with the proteins from emulsions that underwent 24 h inoculation. However, it should be noted that shoulder peaks were observed in the adsorbed proteins extracted from the emulsion with relatively higher addition levels of 13-HPODE or MDA, and with the increase in the addition levels, the intensities of the shoulder peaks became stronger. This may be due to the significant changes in the spatial structures of the adsorbed proteins which caused the exposure of tyrosine residues, and the increasing addition levels of the 13-HPODE or MDA resulted in a higher level of tyrosine residue exposure [39].

### 3.5. SDS-PAGE

**Figure 3:**
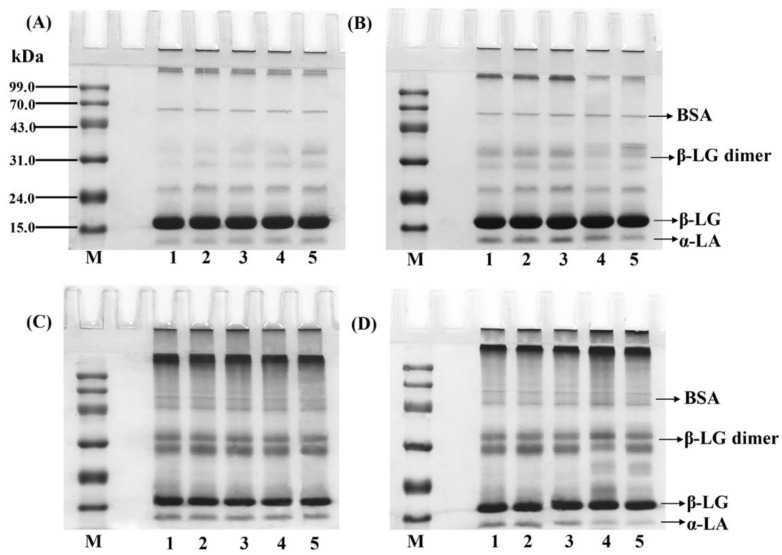
Sodium dodecyl sulfate polyacrylamide gel electrophoresi (SDS-PAGE) of the unadsorbed (**A**,**B**) and adsorbed proteins (**C**,**D**) extracted from O/W emulsions oxidized by different addition levels of HPODE and MDA (Lane M: marker; lanes 1–5: 0, 1, 10, 100, and 1000 μmol/L emulsion) after 24 h incubation. BSA: bovine serum albumin; β-LG: β-lactoglobulin; α-LA: α-lactalbumin.

**Figure 4:**
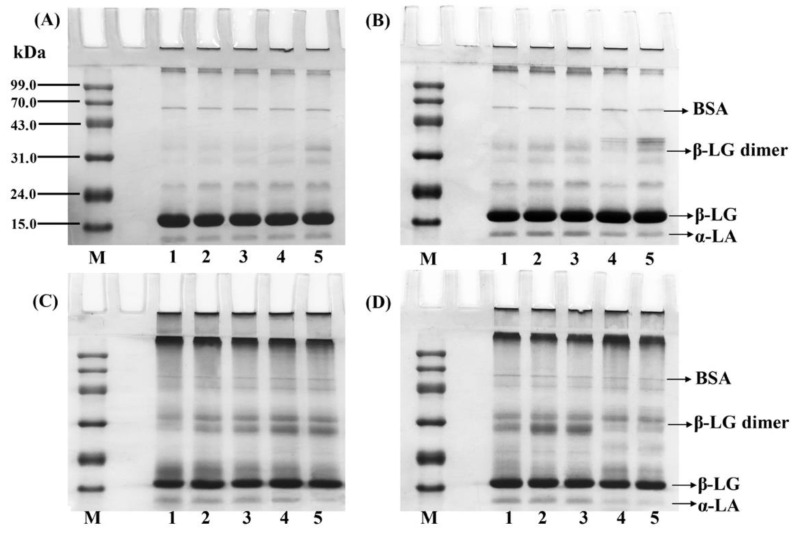
SDS-PAGE of the unadsorbed (**A**,**B**) and adsorbed proteins (**C**,**D**) extracted from O/W emulsions oxidized by different levels of HPODE and MDA (Lane M: marker; lanes 1–5: 0, 1, 10, 100, and 1000 μmol/L emulsion) after 48 h incubation.

Non-denaturing gel electrophoresis is a conventional method to indicate the changes in molecular weights, conformation, and aggregations of proteins [40,41]. To provide further insights into the structural changes of the unadsorbed and adsorbed proteins extracted from the O/W emulsions after 24 and 48 h inoculation, the SDS-PAGE results were analysed as shown in Figure 3 and Figure 4. The WPI proteins contained three primary proteins which can be visibly represented as β-lactoglobulin (β-LG, Mw was 18.4 kDa), α-lactalbumin (α-LA, Mw was 14.2 kDa), and BSA (Mw was 66.2 kDa), and the thin bands around 35 kDa were the β-LG dimer [42]. After 24 h incubation, obvious changes were found in the β-LG dimer of the unadsorbed proteins extracted from the emulsions with 1000 μmol/L MDA. Moreover, several obviously thicker bands of adsorbed protein aggregates appeared on the top of the stacking gel and separation gel, and the band intensity gradually increased with the increasing addition levels of MDA.

After 48 h incubation, clearer changes were found in the β-LG dimer of the unadsorbed and adsorbed proteins extracted from the emulsions with relatively higher levels of MDA. There were also obvious aggregates presented in the adsorbed proteins extracted from the emulsions with relatively higher levels of MDA. The dissociation of β-LG dimers in the adsorbed proteins was more reactive and able to form significant aggregates [41]. Furthermore, an evident disappearance was observed in the α-LA of the adsorbed proteins extracted from the emulsions with 1000 μmol/L MDA. These results proved that MDA may cause more serious unfolding of the unadsorbed and adsorbed proteins compared with the 13-HPODE at relatively higher addition levels. Additionally, Li et al. [19] indicated that the structural changes of soy protein isolate induced by lipid oxidation could obviously increase the droplet size of O/W emulsions during storage, indicated by clear flocculation and creaming. However, due to the higher melting point (33.3 °C) of fully hydrogenated coconut oil [28], droplet size measurement at room temperature (about 22–25 °C) was not conducted in our experiments.

## 4. Conclusions

In summary, the results of our present study, obtained from various analytical techniques, indicated that the structural modifications of WPI in O/W emulsion systems could be initiated and then intensified by primary or secondary LPO products with a concentration-dependent manner and time relationship, respectively. Moreover, compared with primary LPO products (13-HPODE used in our test), the relatively higher amount of secondary LPO products (MDA used in our test) showed a higher degree of oxidative modifications of WPI, implied by the increased protein carbonyl contents and NFK, decreased fluorescence intensity, and the formation of protein aggregation. Additionally, the disparate positions of WPI located in O/W emulsions was another important factor that impacted the degree of oxidative modification. Adsorbed proteins were more readily oxidized by primary or secondary LPO products than unadsorbed proteins. Overall, our results implied that the formation of secondary LPO products was a crucial factor to increase the degree of oxidative modifications of unadsorbed or adsorbed proteins in O/W emulsion systems. Further research will focus on the following two aspects: (1) how to effectively retard the generation of secondary LPO products from primary LPO products, which could probably reduce the oxidative modifications of both unadsorbed and adsorbed proteins, as well as promote the stability of O/W emulsions. (2) determine the oxidative deterioration of both unadsorbed and adsorbed proteins in real WPI-based emulsion foods under prolonged storage conditions (when autoxidation proceeds slowly in terms of both primary and secondary oxidation products), as well as the relative impact on the quality of WPI-based emulsion foods.

## Figures and Tables

**Table 1:** Protein concentrations of unadsorbed and adsorbed proteins extracted from the oil-in-water (O/W) emulsion oxidized by different levels of 13-hydroperoxide (13-HPODE) or malondialdehyde (MDA) for 24 h.

LPO Products	Addition Levels (μmol/L)	Protein Concentrations (mg/mL Emulsion)			
		Unadsorbed Protein	Decreasing Rate (%)	Adsorbed Protein	Increasing Rate (%)
13-HPODE	0	13.21 ± 0.08 ^A^	-	7.13 ± 0.10 ^D^	-
	1	13.09 ± 0.07 ^A^	0.91	7.16 ± 0.06 ^D^	0.42
	10	13.19 ± 0.06 ^A^	0.15	7.17 ± 0.16 ^D^	0.56
	100	12.71 ± 0.12 ^B^	3.79	8.16 ± 0.05 ^C^	14.45
	1000	12.63 ± 0.09 ^B^	4.39	8.27 ± 0.07 ^BC^	15.99
MDA	0	13.14 ± 0.15 ^A^	-	7.23 ± 0.08 ^D^	-
	1	13.10 ± 0.05 ^A^	0.30	7.24 ± 0.10 ^D^	0.14
	10	13.09 ± 0.08 ^A^	0.38	7.28 ± 0.09 ^D^	0.70
	100	12.50 ± 0.14 ^B^	4.87	8.50 ± 0.14 ^AB^	17.57
	1000	12.14 ± 0.08 ^C^	7.61	8.74 ± 0.08 ^A^	20.89

Different uppercase letters (A–D) in the same column indicate significant differences (*p* < 0.05).

**Table 2:** Protein concentrations of unadsorbed and adsorbed proteins extracted from the O/W emulsion oxidized by different levels of 13-HPODE or MDA for 48 h.

LPO Products	Addition Levels (μmol/L)	Protein Concentrations (mg/mL Emulsion)			
		Unadsorbed Protein	Decreasing Rate (%)	Adsorbed Protein	Increasing Rate (%)
13-HPODE	0	13.46 ± 0.13 ^AB^	-	6.30 ± 0.11 ^D^	-
	1	13.53 ± 0.08 ^A^	−0.52	6.40 ± 0.04 ^D^	1.59
	10	13.49 ± 0.09 ^AB^	−0.22	6.49 ± 0.08 ^D^	3.02
	100	13.10 ± 0.05 ^BC^	2.67	7.31 ± 0.05 ^C^	16.03
	1000	13.04 ± 0.07 ^CD^	3.12	7.44 ± 0.10 ^BC^	18.10
MDA	0	13.79 ± 0.10 ^A^	-	6.23 ± 0.09 ^D^	-
	1	13.76 ± 0.10 ^A^	0.22	6.24 ± 0.11 ^D^	0.16
	10	13.65 ± 0.14 ^A^	1.02	6.24 ± 0.13 ^D^	0.16
	100	12.72 ± 0.24 ^D^	7.76	7.87 ± 0.20 ^A^	26.32
	1000	12.69 ± 0.17 ^D^	7.98	7.77 ± 0.16 ^AB^	24.72

Different uppercase letters (A–D) in the same column indicate significant differences (*p* < 0.05).

**Table 3:** Protein carbonyl levels of unadsorbed and adsorbed proteins extracted from the O/W emulsion oxidized by different levels of 13-HPODE or MDA for 24 h.

LPO Products	Addition Levels (μmol/L)	Protein Carbonyl (μmol/g Protein)			
		Unadsorbed Protein	Φ	Adsorbed Protein	Φ
13-HPODE	0	2.32 ± 0.05 ^H^	0.634	5.86 ± 0.05 ^F^	0.979
	1	3.00 ± 0.05 ^G^		5.77 ± 0.04 ^F^	
	10	3.59 ± 0.04 ^F^		5.82 ± 0.04 ^F^	
	100	3.91 ± 0.08 ^E^		6.17 ± 0.07 ^E^	
	1000	4.17 ± 0.07 ^D^		7.05 ± 0.09 ^C^	
MDA	0	3.92 ± 0.18 ^DE^	0.990	6.53 ± 0.09 ^D^	0.999
	1	3.94 ± 0.11 ^DE^		6.59 ± 0.05 ^D^	
	10	4.89 ± 0.08 ^C^		6.66 ± 0.11 ^D^	
	100	7.94 ± 0.07 ^B^		8.77 ± 0.05 ^B^	
	1000	20.52 ± 0.07 ^A^		22.18 ± 0.04 ^A^	

Different uppercase letters (A–H) in the same column indicate significant differences (*p* < 0.05).

**Table 4:** Protein carbonyl of unadsorbed and adsorbed proteins extracted from the O/W emulsion oxidized by different levels of 13-HPODE or MDA for 48 h.

LPO Products	Addition Levels (μmol/L)	Protein Carbonyl (μmol/g Protein)			
		Unadsorbed Protein	Φ	Adsorbed Protein	Φ
13-HPODE	0	2.95 ± 0.05 ^H^	0.795	6.42 ± 0.06 ^G^	0.818
	1	3.50 ± 0.06 ^G^		7.38 ± 0.11 ^F^	
	10	3.85 ± 0.07 ^F^		8.02 ± 0.07 ^E^	
	100	4.30 ± 0.07 ^E^		8.83 ± 0.07 ^D^	
	1000	4.89 ± 0.06 ^D^		10.06 ± 0.05 ^B^	
MDA	0	3.80 ± 0.13 ^F^	0.986	6.65 ± 0.10 ^G^	0.998
	1	4.21 ± 0.06 ^E^		6.66 ± 0.07 ^G^	
	10	6.54 ± 0.09 ^C^		7.23 ± 0.10 ^F^	
	100	8.86 ± 0.04 ^B^		9.42 ± 0.10 ^C^	
	1000	23.77 ± 0.05 ^A^		23.39 ± 0.07 ^A^	

Different uppercase letters (A–H) in the same column indicate significant differences (*p* < 0.05).

**Table 5:** NKF of unadsorbed and adsorbed proteins extracted from the O/W emulsion oxidized by different levels of 13-HPODE or MDA for 24 h.

LPO Products	Addition Levels (μmol/L)	NKF			
		Unadsorbed Protein	Φ	Adsorbed Protein	Φ
13-HPODE	0	54.40 ± 0.20 ^G^	0.906	75.97 ± 0.66 ^F^	0.800
	1	58.49 ± 0.40 ^F^		75.20 ± 0.56 ^F^	
	10	59.55 ± 0.38 ^F^		76.46 ± 0.81 ^F^	
	100	67.24 ± 0.69 ^D^		92.08 ± 0.76 ^D^	
	1000	77.81 ± 1.56 ^C^		97.17 ± 0.63 ^C^	
MDA	0	58.30 ± 0.56 ^F^	0.923	76.94 ± 1.76 ^EF^	0.917
	1	60.29 ± 0.83 ^F^		77.27 ± 0.71 ^E^	
	10	62.63 ± 0.76 ^E^		78.21 ± 1.91 ^E^	
	100	86.27 ± 0.94 ^B^		121.02 ± 0.75 ^B^	
	1000	114.11 ± 0.38 ^A^		164.64 ± 0.87 ^A^	

Different uppercase letters (A–G) in the same column indicate significant differences (*p* < 0.05).

**Table 6:** NKF of unadsorbed and adsorbed proteins extracted from the O/W emulsion oxidized by different levels of 13-HPODE or MDA for 48 h.

LPO Products	Addition Levels (μmol/L)	NKF			
		Unadsorbed Protein	Φ	Adsorbed Protein	Φ
13-HPODE	0	56.09 ± 0.55 ^H^	0.947	76.03 ± 0.69 ^G^	0.801
	1	59.78 ± 0.56 ^G^		76.84 ± 0.72 ^G^	
	10	64.00 ± 0.51 ^F^		87.66 ± 1.25 ^F^	
	100	68.91 ± 0.68 ^E^		114.58 ± 0.90 ^D^	
	1000	87.03 ± 0.80 ^C^		127.30 ± 0.61 ^C^	
MDA	0	58.53 ± 1.17 ^GH^	0.931	75.38 ± 1.53 ^G^	0.898
	1	65.03 ± 1.44 ^F^		76.61 ± 1.20 ^G^	
	10	73.39 ± 0.86 ^D^		95.61 ± 1.29 ^E^	
	100	94.36 ± 1.01 ^B^		135.49 ± 0.74 ^B^	
	1000	133.12 ± 0.84 ^A^		184.74 ± 0.64 ^A^	

Different uppercase letters (A–H) in the same column indicate significant differences (*p* < 0.05).

## Data Availability

The data presented in this study are available in the article.
